# Role of CD4+ T Cells in the Control of Viral Infections: Recent Advances and Open Questions

**DOI:** 10.3390/ijms22020523

**Published:** 2021-01-07

**Authors:** Jérôme Kervevan, Lisa A. Chakrabarti

**Affiliations:** 1 Control of Chronic Viral Infections Group (CIVIC), Virus and Immunity Unit, Institut Pasteur, 75724 Paris, France; jerome.kervevan@pasteur.fr; 2 CNRS UMR, 3569 Paris, France

**Keywords:** CD4+ T cell, antiviral immunity, T cell receptor, virus, HIV controllers

## Abstract

CD4+ T cells orchestrate adaptive immune responses through their capacity to recruit and provide help to multiple immune effectors, in addition to exerting direct effector functions. CD4+ T cells are increasingly recognized as playing an essential role in the control of chronic viral infections. In this review, we present recent advances in understanding the nature of CD4+ T cell help provided to antiviral effectors. Drawing from our studies of natural human immunodeficiency virus (HIV) control, we then focus on the role of high-affinity T cell receptor (TCR) clonotypes in mediating antiviral CD4+ T cell responses. Last, we discuss the role of TCR affinity in determining CD4+ T cell differentiation, reviewing the at times divergent studies associating TCR signal strength to the choice of a T helper 1 (Th1) or a T follicular helper (Tfh) cell fate.

## 1. Introduction

CD4+ T cells orchestrate adaptive immune responses through their capacity to recruit and provide help to multiple immune effectors, in addition to exerting direct effector functions [1]. CD4+ T cells recognize foreign antigens through T cell receptors (TCRs) expressed at their cell surface, and thus maintain the immune system alert against invading pathogens. A single antigen presented by a major histocompatibility complex class II (MHC II) molecule is thought to be sufficient to trigger TCR signaling and CD4+ T cell activation, demonstrating the exquisite sensitivity of this detection system [2]. Mature CD4+ T cells retain a high degree of plasticity, and can differentiate into distinct T helper (Th) types with specialized functions, thus matching the diverse types of encountered pathogens [3]. In the setting of a viral infection, CD4+ T cells differentiate primarily into T helper 1(Th1) effectors, which help cytotoxic CD8+ T cells to lyze infected cells, and into T follicular helper (Tfh) cells, which help B cells to generate highly matured antibodies. CD4+ T cells also establish a dialogue with innate cells, potentiating the functions of NK cells and macrophages through cytokine secretion [4,5]. Activated CD4+ T cells trigger local chemokine production in infected tissues, and thus play a key role in recruiting effector cells to sites of viral replication [6]. Last, highly differentiated antiviral Th1 CD4+ T cells may acquire cytotoxic function and directly lyze infected cells in an MHC II-restricted fashion [7,8].

CD4+ T cells are increasingly recognized as playing an essential role in the control of chronic viral infections [1]. Their importance is best exemplified in human immunodeficiency virus (HIV) infection, where progressive depletion of CD4+ T cells leads to an increased susceptibility to a wide array of pathogens including herpesviruses, polyoma viruses, and papilloma viruses [9]. In this review, we first present recent advances in understanding the nature of CD4+ T cell help provided to antiviral effectors. Drawing from our studies of natural HIV control, we then focus on the role of high-affinity TCR clonotypes in mediating antiviral CD4+ T cell responses. Last, we discuss the role of TCR affinity in determining CD4+ T cell differentiation, reviewing the at times divergent studies associating TCR signal strength to the choice of a Th1 or a Tfh cell fate.

## 2. Rapid Kinetics of CD4+ T Cell Responses in Viral Infections

CD4+ and CD8+ antigen-specific T cell populations expand during the first days to weeks following acute viral infection. CD8+ T cells generally show a greater clonal amplification, as exemplified in vaccination with a live attenuated yellow fever virus [10]. A decline in viremia is usually observed when specific T cells are first detected in the circulation, consistent with a role of these cells in limiting the infected cell population. CD8+ T cells, which are potently cytotoxic and restricted by ubiquitously expressed MHC I molecules, are thought to play a dominant role in the elimination of infected cells at the acute stage of infection. There are some exceptions to this pattern, however, as shown in resolved hepatitis A virus (HAV) infection. HAV-specific CD4+ T cells appear earlier and are amplified to a greater extent than HAV-specific CD8+ T cells, which are rapidly cleared [11]. Specific CD4+ T cell fluctuate in parallel with HAV viremia until the resolution of infection, suggesting a predominant role of the CD4 population in viral clearance. The control of hepatitis C virus (HCV) infection also correlates with the persistence of specific CD4+ rather than CD8+ T cells [12,13]. The immunosuppressive environment characteristic of the liver may dampen the maturation of cytotoxic CD8+ T cells to limit tissue damage, accounting for an important role of CD4 antiviral responses in hepatotropic viral infections. It is also interesting that upon secondary viral infection, such as the one induced by a yellow fever vaccination boost in humans, the kinetics of CD4+ T cell recall response appears faster than that of CD8+ T cells, pointing to the role of CD4+ T cells in ensuring rapid mobilization of immune memory [10]. Mechanistically, MHC II presentation of antigens derived from phagocytosed viral particles may occur more rapidly than MHC I presentation of antigens generated upon productive cell infection [14].

## 3. The Multiple Components of CD4+ T Cell Help Provided to CD8+ T Cells

Classically, CD4+ T cells are thought to help CD8+ T cells indirectly, by licensing antigen-presenting dendritic cells (DCs), which then become more efficient at activating specific CD8+ T cells [1]. The licensing process involves upregulation of CD40 ligand (CD40L) at the surface of CD4+ T cells recognizing their cognate antigen presented by DCs, followed by CD40-CD40L interactions that induce the upregulation of MHC-I and costimulatory molecules CD80/CD86 and CD70 at the DC surface. These bi-directional CD4+ T cell/DC interactions make DCs more efficient at presenting MHC-I-restricted antigens and at activating CD8+ T cells. Licensed DC and activated CD4+ T cells secrete chemokines (CCL3, CCL4, CCL5) that attract CCR5+ CD8+ T cells to sites of antigen presentation, increasing the likelihood of cognate CD8+ T cell encounter [15]. Licensed DCs also produce cytokines such as Interleukin-15 (IL-15) and IL-12 that promote CD8+ T cell survival and differentiation [16]. Furthermore, CD4+ T cell contribute to CD8+ T cell survival via direct CD40L-CD40 interactions and IL-2 production [1]. Helped CD8+ T cells start producing their own IL-2, which limits the induction of the death receptor TRAIL and drives CD8+ T cell proliferation in an autocrine fashion [17]. In terms of transcriptional signatures, CD4+ T cell help induces gene programs that promote CD8+ T cell cytotoxic potential and migratory capacity, while repressing the expression of PD-1 and other co-inhibitory receptors [18]. In addition, CD8+ T cells that receive adequate help at the priming stage are metabolically programmed to become efficient effectors that can mobilize glycolysis upon restimulation [19]. CD4+ T cells also play a key role in the recruitment and maintenance of CD8+ resident memory T cells (TRM) within peripheral tissues, notably through the secretion of the cytokine IFN-γ, which triggers a local production of chemokines involved in mediating CD8+ T cell entry into tissues [20,21].

In the setting of acute viral infections, the induction of an efficient CD8+ T cell response does not always require CD4+ T cell help. Some viruses, such as vesicular stomatitis virus (VSV) and influenza virus, induce sufficient DC maturation to prime antiviral CD8+ T cell in the absence of help [22]. Others, such as the lymphocytic choriomeningitis virus (LCMV) Armstrong strain, require the induction of CD40L on CD4+ T cells to induce optimal CD8+ T cell cytotoxic responses [23]. In general, viruses that induce a strong type I IFN response in acute infection prime CD8+ T cells that show a limited dependency on CD4+ T cell help [24]. In contrast, the role of CD4+ T cell help becomes essential in the setting of recall responses, as CD8+ memory T cells primed in the absence of CD4+ T cell signals are short-lived and show defective responses upon secondary challenge [25]. This is, for instance, apparent in the setting of hematopoietic stem cell transplantation, where delayed reconstitution of the cytomegalovirus (CMV)-specific CD4+ T cell pool associates with an ineffective CD8+ T cell recall response and an increased risk of CMV disease [26,27].

## 4. Defective CD4+ T Cell Help in Chronic Viral Infections Leading to CD8+ T Cell Exhaustion

### 4.1. The “Exhaustion” T Cell Differentiation Program

CD4+ T cells are essential in the setting of chronic viral infections, as demonstrated most clearly in HIV infection, where the progressive depletion of CD4+ helper T cells is accompanied by a progressive dysfunction of CD8+ T cells, which adopt an exhausted phenotype characterized by the expression of multiple inhibitory receptor such as PD-1, TIGIT, and LAG-3, the loss of proliferative and cytokine secretion capacity, and ultimately the loss of cytotoxic activity [28,29]. In contrast, the patients who naturally control HIV infection, called HIV controllers or elite controllers, show highly efficient CD4+ and CD8+ T cell responses (see graphical abstract). In these rare patients, both T cell subsets are characterized by preserved IL-2 secretion, strong proliferative capacity, polyfunctionality (i.e., the simultaneous secretion of multiple cytokines), and cytotoxic capacity predominantly directed at Gag-expressing cells (reviewed in [30,31,32]). Certain of these properties, such as strong proliferative capacity, can be attributed to the low antigenic load present in HIV controllers, which avoids exhaustion through chronic antigenic stimulation and enables central memory T cell differentiation [33]. HIV-specific proliferative capacity can actually be recovered in progressor patients receiving efficient antiretroviral therapy in the long term (>10 years) [34]. In contrast, other properties such as polyfunctionality and potent cytotoxic capacit, do not recover upon treatment, highlighting intrinsic differences between T cells of progressor and controller patients.

In chronic viral infections, T cell exhaustion leads to decreased antiviral function, but can also be viewed as an adaptive mechanism that helps limit tissue destruction in the presence of persisting antigenic stimulation. Recent findings indicate that exhausted CD8+ T cells correspond to a distinct differentiation state, characterized by a specific epigenetic landscape [29]. Exhausted CD8+ T cells can be found in diverse pathological settings such as persistent viral infections but also in the immunosuppressive environment induced by cancerous cells. A flurry of recent studies have highlighted the role of the high-mobility group transcription factor (TF) TOX in maintaining an exhausted state [35,36,37,38,39]. Mechanistically, sustained TCR signaling and unidentified environmental factors lead to calcium-dependent signaling that increases NFAT2 transcription factor (TF) activity in the absence of AP-1 TF activity, resulting in TOX induction. Exhaustion is a gradual process, with a subset of TOX+ CD8+ T cells expressing intermediate levels of PD-1 and maintaining expression of the TF TCF-1, resulting in preserved proliferative capacity. While exhaustion is still reversible in this PD-1-intermediate TCF-1+ population, it becomes fixed and independent of calcium signaling in the PD-1-high population.

### 4.2. Immunotherapy Aimed at “Helpless” Pre-Exhausted Cells

Approaches that aim at rescuing CD8+ T cell from exhaustion by blocking inhibitory receptors, also called immune checkpoint blockade, have revolutionized the field of cancer immunotherapy, but still show very variable efficacy. Studies in the LCMV model suggest that anti-PD-1 antibodies actually act on the PD1-intermediate TCF-1+ subset of pre-exhausted CD8+ T cells, which helps explain the high individual variability in therapy outcome [40,41]. These TCF-1+ pre-exhausted CD8+ T cells share several features with Tfh cells, such as localization in lymphoid tissue and expression of CXCR5, ICOS, and Bcl6, suggesting parallel differentiation programs. Pre-exhausted CD8+ T cells can be detected early in viral infections characterized by persistently high antigenemia, and do not appear to derive from highly differentiated effector cells, raising the possibility that the pre-exhaustion phenotype was acquired at the stage of antigen priming [35]. Considering the striking similarity between the transcriptional signatures of pre-exhausted CD8+ T cells and of CD8+ T cells deprived of CD4+ T cell help, it is proposed that exhaustion is mechanistically linked to the absence of help at the priming stage [18,19]. Thus, strategies that aim at reversing CD8+ T cell exhaustion may benefit from going beyond immune checkpoints blockade, by providing additional components of help, such as costimulatory antibodies or survival cytokines.

## 5. Direct Effector Functions of Antiviral CD4+ T Cells

### 5.1. Antiviral Effect of T Helper 1 (Th1) Cells

Th1 cells exert direct antiviral functions that complement those of the innate and adaptive responses. In particular, activated Th1 cells show an abundant secretion of IFN-γ, a cytokine that activates an array of interferon-stimulated genes (ISG) with intrinsic antiviral activity [42,43]. For instance, Th1 cells were shown to be less susceptible to HIV infection than Th2 cells due to an increased expression of the ISG APOBEC3G, which restricts HIV at the reverse transcription stage [44]. HIV controllers show a predominant Th1 differentiation profile in the HIV-specific CD4+ T cell pool, consistent with a protective effect of this particular subset [45,46]. It should be noted, however, that inborne errors in the IFN-γ pathway in humans lead primarily to an increased susceptibility to mycobacteria, rather than to viruses, suggesting a degree of redundancy in pathways involved in antiviral immunity [47].

### 5.2. Antiviral Effect of Cytotoxic CD4+ T Cells

In vitro models have shown that Th1 cells subjected to chronic antigenic stimulation in the presence of IL-2 differentiate into cytotoxic CD4+ T cells capable of lyzing antigen presenting cells (APC) in an MHC II restricted fashion [8]. These cells acquire perforin and granzyme-dependent lytic capacity, while losing CD27/CD28 costimulatory molecule expression. IL-2 secretion capacity is also lost, while production of effector cytokines such as IFN-γ and TNF-α can remain efficient. This differentiation program depends on a shift in the ThPOK/Runx3 TF balance that promotes the acquisition of CD8+ T cell characteristics while maintaining CD4+ T cell expression [7]. Other pathways for cytotoxic CD4+ T cell generation likely exist, as suggested by the existence of a small CD4+ T cell subset expressing the CRTAM adhesion molecule that preferentially gives rise to the CD4+ cytotoxic T cell lineage in mice [48].

Animal model studies have confirmed the induction of cytotoxic CD4+ T cells in a variety of viral infections, often with a preferential localization in the lung or gut mucosa [8]. For instance, cytotoxic CD4+ T cells contribute to the clearance of influenza A virus (IAV) in the lung, as suggested by the perforin-dependent decrease in viral load and the emergence of MHC-II restricted IAV epitope mutants observed upon memory CD4+ T cell transfer [49]. In humans, cytotoxic CD4+ T cells reach high frequencies during the acute stage of the infection induced by vaccinia virus [50]. Cytotoxic CD4+ T cells are also abundant in human CMV infection, and are thought to contribute to viral containment through polyfunctional cytokine secretion combined with direct cytotoxicity [51,52]. In contrast, newborns with congenital CMV infection and persistent viral shedding show defective CD4+ and CD8+ cytotoxic T cells with limited cytokine secretion capacity and signs of immune exhaustion [53]. Cytotoxic CD4+ T cells also emerge during the acute stage of HIV infection, but appear to be rapidly lost at the chronic stage, consistent with the preferential targeting of activated CD4+ T cells by HIV [54,55,56]. Of note, a longitudinal study showed that patients with a higher frequency of HIV-specific CD4+ T cells with cytotoxic potential (granzyme A+) at the acute stage reached lower viral replication setpoints at the chronic stage [57]. Furthermore, natural HIV controllers maintained a population of HIV-specific CD57+ CD4+ T cells with lytic granule markers in the long term, raising the possibility that cytotoxic CD4+ T cells contributed to HIV control [58].

## 6. Immunoregulation in Chronic Viral Infections

Continuing cytotoxic activity due to viral antigen persistence clearly poses a risk of tissue damage. For instance, in CMV infection, expression of the fractalkine CX3CR1 receptor at the surface of cytotoxic CD4+ T cells was proposed to recruit these cells to the activated vascular endothelium, possibly contributing to the induction of inflammatory vascular disease [52]. More broadly, chronic T cell activation has deleterious consequences in the long term. Persisting T cell activation in HIV infection is thought to accelerate immunoscenescence and contribute to the loss of adaptive responses to opportunistic pathogens [59].

### 6.1. Upregulation of Inhibitory Receptors

Multiple immunoregulatory mechanisms wired into T cell differentiation programs mitigate the effects of persisting immune activation. Chronic TCR stimulation induces a T cell exhaustion program, which also applies to CD4+ T cells. The few HIV-specific CD4+ T cells that persist in chronic HIV infection express an array of inhibitory receptors and show defective proliferative and cytokine secretion capacity [60,61]. Dysfunction persists even after the control of HIV viremia through antiretroviral therapy, suggesting that T cell exhaustion is not fully reversible. CD4+ T cells that coexpress multiple inhibitory receptors (PD-1, TIGIT, LAG-3) preferentially harbor latent HIV proviruses and, thus, constitute a major part of the HIV reservoir in treated patients [62]. This poses an obstacle to HIV eradication strategies that aim at purging latently infected cells through reactivation, as exhausted cells reactivate poorly.

### 6.2. Interleukin-10 (IL-10)-Dependent Suppression by Tr1 Cells

Another immunoregulatory mechanism lies in the induction of the cytokine IL-10, which dampens the function of multiple immune effectors. IL-10 induction is wired into the Th1 differentiation program, as chronically activated Th1 cells naturally lose IFN-γ expression while upregulating IL-10 under the influence of the TF Blimp-1 [63]. Of note, HIV-specific CD4+ T cells from treated patients show a more frequent induction of IL-10 upon restimulation than those of natural HIV controllers, suggesting again that control of antigenemia through antiretroviral therapy was not sufficient to relieve negative immunoregulation [45]. Persisting immunoregulatory conditions such as TCR stimulation in an environment rich in IL-10 and IL-27 lead to the induction of specialized CD4+ regulatory T cells called Tr1 [64]. These cells mediate immunosuppression through multiple mechanisms including IL-10 and TGF-β secretion, granzyme-dependent cytotoxic activity directed at APC, and conversion of the inflammatory mediator ATP to adenosine via the ectoenzymes CD39 and CD73. Tr1 cells were proposed to antagonize excessive immune activation in primary HIV infection [61]. High frequencies of Tr1 cells were also detected in HCV-infected patients who did not spontaneously clear the virus, raising the possibility that negative immunoregulation contributed to viral persistence [65].

### 6.3. Regulatory T Cell (Treg)-Dependent Immunoregulation

Tregs generated in the thymus and in the periphery constitute a CD4+ T cell subset dedicated to the control of immune activation, and as such play a key role in limiting immunopathological damage in viral infections [66]. Tregs are characterized by the TF FoxP3 and typically express the IL-2 receptor-alpha chain at high levels (CD25hi) and the IL-7 receptor-alpha chain at low levels (CD127lo). Tregs suppress T cell responses through a variety of mechanisms that overlap with those used by Tr1 cells. In addition, Tregs capture IL-2 with high affinity through CD25, thus depriving neighboring T cells of this key cytokine and efficiently limiting T cell proliferation. Tregs effectively limit immunopathology in chronic and recurring viral infections, as shown for instance in a murine model of influenza virus infection, where a secondary challenge in the absence of memory Tregs led to letal pulmonary inflammation [67].

Recently, two Tregs subsets were identified based on differential expression of immunosuppressive cytokines: IL-35 producing Tregs were mostly involved in limiting T cell activation within the T cell zone of lymphoid organs, while IL-10 producing Tregs migrated to inflamed peripheral tissues to exert a broader immunosuppressive effect on both APCs and T cells [68]. IL-35 functions primarily by promoting Treg differentiation at the expense of effector T cell differentiation [69]. Increased IL-35 expression in chronic HBV infection is thus thought to promote Treg function and to facilitate viral persistence [70]. The role of Tregs in HIV infection remains debated, with evidence for both a beneficial role in dampening chronic immune activation and a detrimental role in suppressing antiviral T cell responses (reviewed in [66]). The negative costimulatory molecule CTLA-4, which plays a key role in Treg function, is expressed proportionally to the viral load in HIV-specific CD4+ T cells, and may thus contribute to immune dysfunction in uncontrolled HIV infection [60]. In the case of murine Friend virus infection, Treg amplification is marked at the acute stage and is responsible for an increased viral load [71]. Thus, Tregs play an equivocal role in viral infections, as the balance between protection from immunopathology and facilitation of viral persistence depends on the pathogen and the infection setting.

### 6.4. Perturbed Treg/Th17 Balance in Chronic HIV Infection

Th17 effector cells and Tregs can both differentiate in environments enriched in TGF-β such as mucosal tissues. The proportions of the two subsets is regulated by the status of neighboring APC and the local concentration of inflammatory mediators. For instance, in inflammatory conditions, the enzyme indoleamine 2,3-dioxygenase (IDO) expressed by certain DC subsets generates tryptophan metabolites that stabilize FoxP3 expression, resulting in a shift of the Treg/Th17 balance towards an increased proportion of Tregs [72]. HIV and SIV preferentially deplete activated Th17 cells in the intestinal mucosa, resulting in a relative increase of the Treg subset [73,74]. This does not efficiently limit mucosal inflammation, however, as Th17 are needed to maintain intestinal epithelial integrity through IL17 and IL-22 secretion. In a situation of Th17 depletion, a compromised epithelial barrier becomes permissive to microbial translocation, which further drives inflammation and enterocyte damage [75]. The Th17 population does not fully recover under antiretroviral therapy, due to the establishment of a Th1-like proinflammatory environment in the intestinal mucosa [76]. In particular, increased secretion of IFN-γ and IL-18 antagonize the synthesis of the CCL20 and CCL25 chemokines by intestinal epithelial cells, resulting in an inhibition of Th17 cell recruitment through CCR6 and CCR9, respectively. Therefore, HIV infection not only depletes activated CD4+ T cells but also alters the differentiation program of surviving CD4+ T cells, resulting in long-term immune dysfunction.

## 7. Shifting Th1/T Follicular Helper (Tfh) Balance in Chronic Viral Infections

Although historically the focus was placed on Th1 cells, it is now well established that Tfh cells are also required for the control of viral infections [77]. Tfh cells are needed for the development of germinal centers in lymphoid organs, where they interact with maturing B cells that undergo somatic hypermutation and class-switch recombination. This interaction is at the core of the selection process that promotes the survival of high-affinity antibody-secreting plasmablasts and memory B cells. Ablation of Tfh cells in mice models of LCMV and vaccinia virus infection causes a decrease in the amount and quality of antiviral antibodies, resulting in limited virus neutralization capacity and increased viral persistence [78,79]. In humans, the frequency of activated Tfh cells in the circulation following flu vaccination predicts the development of neutralizing antibodies [80,81].

### 7.1. Multiple Parameters Control Tfh Differentiation

Tfh cells are characterized by a high expression of PD-1, of the CXCR5 chemokine receptor, which drives homing to germinal centers, and of the IL-21 cytokine, which provides survival signals to B cells [77]. Recent findings indicate that PD-1 expression helps confine Tfh cells to the germinal center, by repressing expression of the chemokine receptor CXCR3, which would drive Tfh cell emigration [82]. Tfh differentiation depends on expression of the master TF regulator Bcl-6, which acts in concert with other TF such as c-Maf, IRF4, Ascl-2, BATF, and TCF-1 to confer full Tfh function [77]. An important regulatory loop between Bcl-6 and Blimp-1, which repress each other, determines the balance between Tfh and other CD4+ effector cell differentiation [83]. The cytokine IL-2 is a potent inductor of Blimp-1 and drives the differentiation of Th1 cells; conversely, IL-6 produced by B cells promotes Bcl-6 expression and Tfh differentiation in murine models. In human cells, IL-2 does block Tfh differentiation, while IL-12, rather than IL-6 may promote Tfh differentiation [84]. The role of the TF T-bet is ambivalent in mice, as it drives Th1 differentiation, but is also a negative regulator of IL-2, and hence helps stabilize the Tfh phenotype [85]. This may explain why a transient expression of T-bet early in Tfh differentiation is required for the optimal development of germinal center responses in the LCMV infection model. Tfh differentiation is also finely regulated at the spatial level through specialization of distinct niches in lymphoid tissues. For instance, activated mouse CD4+ T cells expressing the G-protein coupled receptor EBI-2 relocalize to the T cell zone/follicle interface, where they are exposed to CD25-expressing DC that scavenge IL-2, resulting in preferential Tfh differentiation [86]. Recent findings have also emphasized the importance of rapid and localized type I IFN induction in the lymphoid priming niche for Tfh differentiation [87]: the cytopathic virus VSV induced an early (8 h) IFN-α peak in the T cell zone, leading to rapid IL-6 expression in DC, which primed Tfh differentiation; in contrast, the less cytopathic virus LCMV induced a delayed (24 h) IFN-α peak, a lower IL-6 response from DC, and an initially predominant Th1 response. Thus, the Th1/Tfh balance is regulated through spatial but also temporal variations in the cytokine microenvironment.

### 7.2. Preferential Expansion of Tfh Cells in Chronic Viral Infections

**Figure 1:**
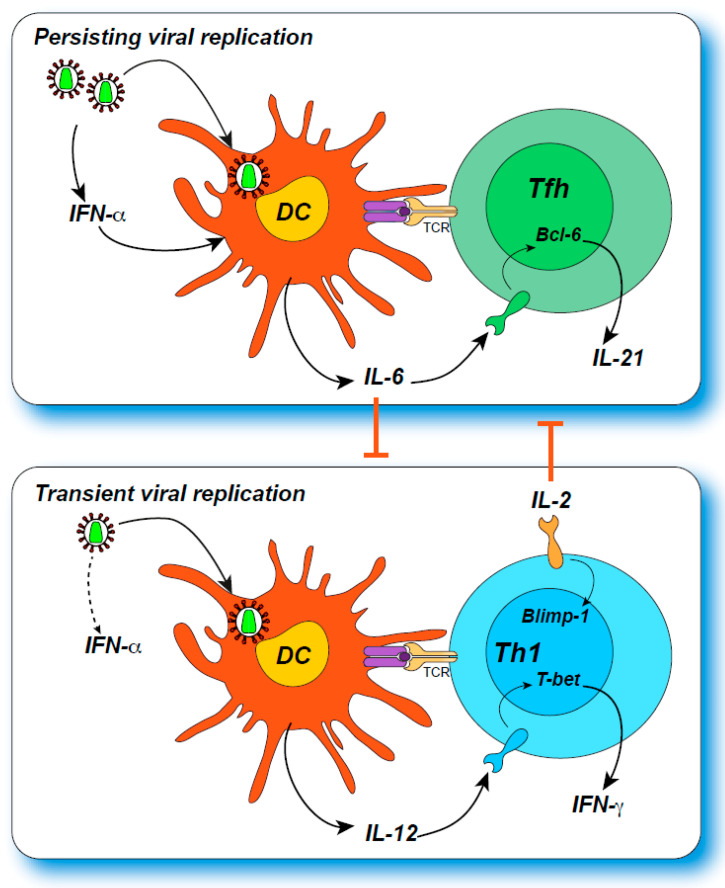
The cytokine environment regulates the Tfh/Th1 balance. **Top panel**: Upon prolonged or inflammatory viral replication, IFN-α production inducesIL-6 secretion by dendritic cells, which promotes Tfh differentiation through Bcl-6induction. Tfh cells then produce IL-21, which provides help to maturing B cells. **Bottom panel**: Transient and non-cytopathic viral replication induces limited IFN-αproduction but still causes DC maturation with IL-12 production, which promotes Th1differentiation via T-bet induction. IL-2 also plays a key role in Th1 differentiation by inducing Blimp-1 and inhibiting Tfh differentiation. Reciprocally, IL-6 inhibits Th1differentiation, in part through downregulation of the IL-2 receptor.

In several models of viral infection [78,88,89,90], persisting viremia leads to a progressive amplification of Tfh responses at the expense of Th1 responses (Figure 1). This can be viewed as an adaptation to limit immunopathology in the face of an initially unsuccessful antiviral response, as antibody-mediated virus neutralization may still limit viral spread without causing inflammatory or cytotoxic tissue damage.

In the LCMV model, persisting antigen signaling through the TCR accompanied by a late increase in IL-6 secretion drives Tfh cell amplification [88,89]. The progressive inhibition of Th1 differentiation depends in part on type I IFN signaling, which is sustained by viral persistence [91]. An early induction of the TF TCF-1, which shifts the Bcl-6/Blimp-1 balance towards Bcl-6 expression, marks a subset of CD4+ T cells for Tfh differentiation [92]. It is interesting to note that TCF-1 expression characterizes both pre-exhausted CD8+ T cells and CD4+ T cells committed to the Tfh lineage [92,93], suggesting shared pathways for adaptation to antigen persistence in both populations. Recent findings suggest that, paradoxically, CD4+ T cells fated to become Tfh are characterized by an abundant production of IL-2, but a poor responsiveness to this cytokine [83]. Mechanistically, IL-6 appears to inhibit expression of the IL-2 receptor beta-chain, allowing activated CD4+ T cells to produce IL-2 without receiving an autocrine signal that would promote Th1 differentiation [94]. The nature of cytokines involved in shifting the Th1/Tfh balance may vary depending on the tissue considered. For instance, TGF-β signaling in the lung mucosa downregulates CD25, making influenza-specific CD4+ T cells less sensitive to IL-2 signaling and driving them towards the Tfh lineage [95]. Thus, multiple mechanisms converge in inhibiting IL-2 signaling to drive preferential Tfh amplification. Of note, Tfh differentiation may be inhibited, rather than promoted, in certain types of acute viral infection. This could be the case for instance in patients with a fatal form of COVID-19, who showed a striking lack of germinal centers in thoracic lymph nodes and spleen obtained at autopsy [96]. In these cases, inhibition of Tfh differentiation was ascribed to excessive TNF-α production, illustrating the risks associated to an excessive inflammatory response. Patients with fatal COVID-19 still seroconverted but showed signs of extrafollicular rather than germinal center (GC)-dependent B cell responses. As plasma cells generated at extrafollicular sites tend to be short-lived [97], there is a risk that the antibody response to severe COVID-19 may rapidly wane. This does not mean, however, that SARS-CoV-2 specific responses induced by vaccines would not be long-lasting, as they would be primed in a very different inflammatory context.

### 7.3. Pertubed Tfh Function in Progressive HIV Infection

The progressive shift towards Tfh differentiation in chronic viral infection explains the often delayed emergence of neutralizing antibodies which, nevertheless, ensures viral control in the LCMV model [78]. Complex Tfh cell/virus interactions take place in chronic HIV infection, as the virus preferentially targets the Tfh population, leading to a particularly delayed and inefficient neutralizing antibody response [98]. As HIV infection progresses towards the chronic stage, patients do show signs of increased Tfh differentiation, as indicated by hyperplasic germinal centers, lymphadenopathy, and hypergammaglobulinemia. In addition, the frequency of HIV-specific Tfh cells is increased as compared to other HIV-specific CD4+ T cell subsets in lymphoid tissue [90]. At the same time, HIV efficiently replicates in the Tfh population, and establishes a preferential reservoir in this subset as measured by proviral copy number [99]. Susceptibility of the Tfh subset to infection is facilitated by the activated status of these cells, and by Bcl-6 mediated repression of ISG that usually suppress HIV, such as MX2 and IFITM3 [100]. Tfh function is impaired in viremic patients, in part through increased PD-1/PD-L1 inhibitory interactions within germinal centers [101]. In contrast, circulating HIV-specific Tfh cells from HIV controllers appear to maintain a high degree of functionality [102,103]. Preserved Tfh function in controllers is associated to a higher frequency of circulating HIV-specific memory B cells [104,105]. Most HIV viremic patients fail to develop efficient neutralizing antibodies even after years of chronic infection, one main reason being the continuous selection of escape mutations in the viral envelope [106]. In rare cases, persisting Tfh function enables the continuous selection of neutralizing antibodies that match successive mutant viral strains, resulting in the generation of broadly neutralizing antibodies (bNAbs). The virus usually remains one step ahead in this evolutionary race, so that bNAbs do not induce viral control in patients who develop them. However, the capacity of cloned bNAbs to target a majority of HIV strains make them promising tools for HIV treatment and prevention, as exemplified by recent clinical trials [107].

## 8. Influence of TCR Affinity on T Cell Function and Dynamics

The strength of signals received through the TCR determines in large part CD4+ T cell activation, acquisition of effector functions, proliferation, and survival [108,109,110,111]. TCR signal strength is in turn determined by antigen availability and by the intrinsic affinity of the TCR for peptide-MHC (pMHC) complexes. The nature of the biophysical parameter that best correlates with T cell activation potency has been debated, with models based on binding affinity or on the dissociation kinetics of TCR/pMHC complexes [112]. The development of affinity measurements in 2D, where TCR and pMHC are anchored into lipidic membranes to better approach physiological conditions of T cell activation, suggests that an agonist pMHC can bind the same TCR serially with rapid kinetics, and that the aggregated half-life of the interaction is a better predictor of ligand potency [113,114].

A convergent picture of the dynamics of the antigen-specific T cell repertoire during infection has emerged from multiple infection and immunization studies [115,116,117,118]. In polyclonal T cell responses, high-affinity TCR clonotypes proliferate more efficiently, and are enriched at peak response. However, differential susceptibility to activation-induced cell death, exhaustion and/or anergy during the contraction phase of the response decrease high-affinity clonotype frequency, so that the diversity of the overall TCR clonotypic repertoire is maintained. Upon recall responses, amplification of high-affinity memory clonotypes is even more marked, so that the bulk of the effector response is often dependent on a few dominant high-affinity clonotypes. A relative return to clonotypic diversity is again observed upon antigen elimination, although dominant memory clonotypes persist at a higher frequency than in a naive repertoire.

Although the key features of this model of TCR repertoire evolution remain valid, a more nuanced picture has emerged from recent studies. While MHC tetramer binding assays predominantly used in early studies detect only cells expressing a TCR of relatively high affinity (with a Kd lower than 80 µM for MHC I binding as measured by 3D surface plasmon resonance), the more recent 2D pipette adhesion assays detect cells with a broader range of TCR affinities [113,119]. It is now apparent that T cells of low affinity are more abundant than previously thought and can make a significant contribution to antigen-specific responses [120,121]. Furthermore, changes in the nature of APC can influence the repertoire of responding T cells. For instance, antigen specific B cells clonally amplified in the course of Friend virus infection capture viral antigen with very high affinity through their BCR and thus present sufficient antigenic peptides to activate low affinity T cells, resulting in a second wave of responding CD4+ T cells with a broader TCR repertoire than observed in the first wave [122]. In addition, CD4+ T cells with equivalent TCR affinity for a foreign antigen may respond differentially, based on a variable capacity for self recognition. This is because the degree of a TCR cross-reactivity for self-antigen sets a level of tonic signaling that conditions not only the survival of naive CD4+ T cells, but also the strength of their response upon encounter with foreign antigen [123]. Thus, intrinsic TCR parameters influence but are not sufficient to define CD4+ T cell potency.

## 9. The Role of TCR Signal Strength in Viral Control

### 9.1. Role of TCR Affinity in Antiviral CD8+ T Cell Potency

It has long been recognized that amplification of high-affinity TCR clonotypes in antiviral CD8+ T cells is associated with the control of chronic viral infections, as shown for instance in animal models of infection by vaccinia virus, herpes simplex virus-1, equine infectious anemia virus, paramyxovirus simian virus-5, and respiratory syncytial virus (RSV) [124,125,126]. In contrast, low-affinity TCR clonotypes accumulate in persisting viral infections, as shown for instance during the phase of memory CD8+ T cell inflation observed in CMV infection [127]. TCR clonotypic analyses in humans point to the amplification of high-affinity clonotypes in controlled HCV and HIV infections [128,129,130,131]. The role of high-affinity TCR clonotypes in HIV control remains debated, however, as CD8+ T cells harboring such clonotypes are more prone to immunosenescence and exhaustion [132,133]. Antiviral memory CD8+ T cells from controllers are proposed to adopt an exhaustion-resistant metabolic profile independently of TCR affinity [134]. In addition, genetically variable viruses such as HIV can escape high-affinity TCR recognition through epitope mutation [135]. For this reason, cross-reactive TCRs capable of recognizing both the wild-type and mutated versions of an epitope may play a predominant role in HIV control [136,137]. The capacity of HIV-specific CD8+ T cells to suppress viral replication in infected target cells directly correlates with their sensitivity to antigen [138,139], and that CD8+ T cell with higher TCR affinity show a better ability to reduce the HIV reservoir in reactivated CD4+ T cells [140]. Thus, “shock and kill” strategies that aim at decreasing the HIV reservoir through reactivation followed by elimination of infected cells through cytotoxic activity will require the presence of high-affinity TCR clonotypes in the effector CD8+ T cell population [141].

### 9.2. Role of TCR Affinity in CD4+ T Cell Helper and Antiviral Functions

TCR clonotypic analyses in antiviral CD4+ T cells have been comparatively fewer, in part due to technical issues in detecting those cells. Compared to CD8+ T cells, CD4+ T cells generally show a lower burst size and lower overall TCR affinity, making their detection by MHC II tetramer labeling challenging. However, improvements in MHC tetramer technology have enabled the detection of rare cells, and shown for instance that the precursor of antiviral CD4+ T cells may acquire a memory phenotype through cross-recognition of environmental or self antigens, thus priming CD4+ T cells for future infections [142,143]. The influence of TCR affinity on CD4+ T cell expansion could be documented in several models. For instance, the response to the Friend virus in mice was dominated by high-avidity CD4+ T cells expressing a given TCR Vα chain [144,145]. In that particular case, expression of endogenous retroviruses with homologies to Friend virus shaped the TCR repertoire by negative selection, but the CD4+ clonotypes that escaped selection were sufficiently potent to help control Friend virus infection. In RSV infection, TCR affinity also influences the hierarchy of CD4+ T cell clonotype amplification. Comparison of effector and regulatory CD4+ T cells recognizing the same RSV epitope showed that effector cells were of higher avidity, and were preferentially amplified upon rechallenge, leading to a more potent secondary response [146]. Of note, high-affinity CD4+ T cell priming in the presence of strong adjuvants can lead to unexpectedly stunted recall responses. This was shown for instance in a model of influenza virus vaccination, where high-affinity peptide priming in the presence of a TLR4 ligand led to the generation of highly differentiated Th1 effector cells with limited proliferative capacity, contrasting with the higher magnitude recall response obtained after low-affinity peptide priming [147]. Recent findings indicate that high-affinity CD4+ T cells play an important role in the persistence of CD8+ TRM cells in peripheral tissues, as shown in mouse polyoma virus infection [148]. In this model, high-affinity CD4+ T cells are preferentially recruited to the infected brain, where they adopt a mixed Th1/Tfh differentiation and produce IL-21, which is required for the persistence of TRM CD8+ T cells. A similar requirement for help may apply to human JC polyomavirus infection, explaining why this virus causes progressive multifocal leukoencephalopathy, a devastating demyelinating disease, in situations of CD4+ T cell loss, such as AIDS and idiopathic CD4 lymphocytopenia [149].

### 9.3. High-Affinity Public TCR Clonotypes Sustain an Efficient CD4+ T Cell Response in Controlled HIV Infection

In the case of HIV infection, virus-specific CD4+ T cells are lost early in viremic patients due to preferential depletion of these cells by the HIV [150,151]. HIV controllers, in contrast, maintain a sizable HIV-specific CD4+ T cell population, which is preferentially directed at Gag rather than Env antigens [152]. We could show that Gag-specific CD4+ T cells from natural HIV controllers contained a high-avidity subset with high proliferative capacity. In contrast, the few HIV-specific CD4+ T cells that persisted in patients who received antiretroviral therapy retained only a low TCR avidity, as measured by MHC II tetramer staining [153]. TCR sequence analysis of CD4+ T cells specific for the most immunodominant epitope in HIV-1 capsid, Gag293, revealed the presence of public clonotypes preferentially shared by HIV controllers [154]. Transfer of one of these public TCRs to healthy donor cells was sufficient to confer a series of properties characteristic of controller CD4+ T cells, including high antigen sensitivity and polyfunctional cytokine secretion capacity. Furthermore, public TCRs from controllers also conferred MHC II-restricted cytotoxic capacity, suggesting that CD4+ T cells could directly contribute to HIV control [155]. Cytotoxic capacity was only observed for the TCRs of highest affinity, emphasizing the functional importance of the high-affinity subset among HIV-specific CD4+ T cells. Of note, high TCR affinity also correlated with broad HLA II cross-restriction, with a single TCR recognizing the Gag293 epitope presented by up to 5 distinct HLA-DR alleles. From a structural standpoint, this TCR mediated a peptide-centric recognition of the Gag293-MHC II complex, explaining how it could tolerate mismatches in MHC II [155]. HLA II cross-restriction helped explain how public TCRs could be shared by HIV controllers with different HLA II genotypes. Class II cross-restriction may also explain why biases in the representation of HLA II alleles remain limited (although detectable) in the HIV controller population, while HLA I biases are prominent [156,157]. Taken together, these studies pointed to the role of high TCR affinity in shaping the efficient CD4+ T cell responses in controlled HIV infection. The fact that such responses remain undetectable in non-controller patients even after decades of antiretroviral therapy suggests that the quality of the HIV-specific TCR repertoire does not recover even though CD4+ T cells can return to near normal levels. This poses a challenge for strategies that aim at therapy interruption, and suggests that TCR transfer approaches or de novo T cell priming may be required to establish an immune-mediated control of HIV.

## 10. Influence of TCR Affinity on T Helper Differentiation in Viral Infections

### 10.1. Regulation of the Th1/Th2 Balance

The strength of TCR signals was shown early on to influence CD4+ T cell helper differentiation, even though the influence of the cytokine environment was also well recognized. It is generally agreed that weak TCR signals promote Th2 differentiation, while stronger signals drive Th1 differentiation [158]. The strength of TCR signals integrates both the intrinsic TCR affinity and the amount of available antigen presented at the APC surface. At the molecular level, TCR signal strength is reflected in the level of the TF IRF4, which associates with the TF BATF to bind enhancers of different sensitivities to the IRF4-BATF complex, resulting in the differential induction of genes important in T helper differentiation [159]. Of note, TCR signal strength controls the induction of cytokine receptors such as IL-12R, and thus influences the sensitivity of recently activated CD4+ T cells to the cytokine milieu [160].

### 10.2. Regulation of the Th1/Tfh Balance

How TCR signal strength influences the Th1/Tfh balance has remained a vexing question. Both subsets are clearly induced in the context of viral infections, but often with different kinetics and in different proportions. TCR clonotypic analyses of flu-specific Tfh and Th1 cells in humans indicate that the two subsets are clearly distinct, with few shared clonotypes [161]. Early studies in mouse models suggested that CD4+ T cells expressing a high-affinity transgenic TCR preferentially differentiated into Tfh cells [162]. Tracking the fate of single naive CD4+ T cells after adoptive transfer showed that (i) the progeny of a single cell could adopt different T helper phenotypes and (ii) that the proportion of Th1, Tfh, and germinal center Tfh (GC Tfh) cell progeny depended on the nature of the TCR expressed by the original cell [163]. In this system, TCR with very long dwell times yielded a higher proportion of Tfh and GC-Tfh, possibly because of activation-induced cell death in the Th1 subset. Another extensive analysis of single CD4+ T cell fate confirmed that identical naive CD4+ T cells could take different fate decisions (central memory TCM, Th1 effector, or Tfh) in a probabilistic manner, and reported that overall stronger TCR signals were required for the differentiation of Tfh cells [164]. A recent study in an IL-2 reporter mice model suggested that TCR transgenic CD4+ T cell receiving the strongest signals produced more IL-2, but paradoxically remained insensitive to IL-2 signaling, possibly due to lack of CD25/IL2-Rα upregulation, resulting in a preferential Tfh differentiation [83]. On the other hand, a series of studies support the idea that strong TCR signals lead to sustained expression of the high-affinity IL-2R and promote Th1 differentiation [165,166]. Transcription factor analyses also report a direct link between strong TCR signals, high IRF4 expression, and Th1 effector differentiation [167]. Interestingly, a recent study may help reconcile these divergent findings: in the LCMV infection model, Künzli et al. reported that TCR signal strength exerted opposing effects on CD4+ T cells responding to acute versus chronic viral infection [168]. To test this notion, the authors generated a series of mutations altering the gp61 epitope recognized by the SMARTA TCR, and introduced these mutations into both the Armstrong and clone 13 LCMV strains. During acute infection with the Armstrong strain, viruses with a strong epitope led to preferential differentiation and amplification of Th1 cells. In contrast, during LCMV clone 13 infection with persistently high antigenemia, viruses expressing a strong epitope preferentially induced the amplification of Tfh cells. These findings highlight the importance of viral antigen persistence, which rewires the T cell differentiation process, possibly through the selective death or exhaustion of over-stimulated Th1 cells, or through the influence of virally induced cytokines such as type I interferons and IL-10 [169,170,171]. The reorientation of high-avidity CD4+ T cells away from a Th1 effector cell fate likely limits cytotoxicity mediated by both CD4+ and helped CD8+ T cells. Taken together, several mechanisms converge in limiting immunopathological damage in chronic viral infections, including favored Tfh differentiation, Treg/Tr1 induction, and ultimately T cell exhaustion. These mechanisms may be viewed as relevant adaptations towards disease tolerance in situations of persistent viremia [172], and may have to be tackled together in immunotherapeutic approaches that aim at restoring viral control.

## 11. TCR Clonotypic Analyses Shed New Light on the Dynamics of Antiviral Responses

Improvements in deep sequencing technology coupled to the development of single cell methods for paired TCR chains amplification have enabled in depth studies of virus-specific TCR repertoires. TCR diversity emerged as an important parameter, for instance in human CMV infection, where a broader clonotypic repertoire in tetramer+ CD8+ T cells associated with signs of lower viral replication [173]. In the context of HIV infection, the loss of TCR repertoire diversity in both the CD4+ and the CD8+ T cell populations was also associated with disease progression in early studies [174,175]. TCR clonotypes are now being used as molecular markers to track the amplification of latently infected CD4+ T cell clones, demonstrating that HIV provirus persistence depends largely on homeostatic CD4+ T cell proliferation [176]. Interestingly, a subset of latently infected cells were shown to be specific for HIV and CMV, suggesting that viral reactivation episodes could also drive the amplification of the HIV reservoir [177,178].

TCR clonotypic analyzes are also of interest in vaccination studies, to track the induction and long-term persistence of vaccine-specific T cells. Influenza virus vaccination, for instance, was shown to induce an oligoclonal population of circulating Tfh cells that had signs of activation (CD38+ ICOS+) at day 7 post-vaccination, that persisted over years as memory Tfh cells, and that could be reactivated upon yearly revaccinations [179]. An HIV DNA vaccine was shown to induce a subset of TCR clonotypes also shared by CD4+ T cells of HIV controllers, suggesting the potential for high-avidity CD4+ T cell responses [180]. In a therapeutic vaccination trial directed at varicella zoster virus (VZV), the vaccine did not boost the dominant VZV-specific CD4+ T cell clones, but rather stimulated subdominant or naive CD4+ T cell clones, pointing to a diversification of the antiviral clonotypic repertoire [181].

Large-scale TCR sequencing studies now rely on algorithms and preexisting clonotype databases to attempt predicting TCR specificity. Common CDR3 motifs could often be identified in TCRs specific for a given immunodominant viral epitope, leading to a better definition of residues driving TCR recognition [182,183]. While structural studies are still needed to precisely define TCR specificity [184], bulk analyses of TCR sequences are starting to shed light on individual immune history [185]. It is interesting for instance that bulk TCR-β sequencing performed on the blood of COVID-19 patients identified motifs enriched exclusively in recovered patients, opening the door to the identification of protective TCR clonotypes [186].

## 12. Concluding Remarks

Recent advances have emphasized the importance of CD4+ T cell help in the maintenance of antiviral CD8+ T cells in a non-exhausted state, and in the persistence of TRM CD8+ T cells in a tissue-specific manner. The direct cytotoxic function of CD4+ T cells has been confirmed in several models of viral infections, and shown to depend tightly on TCR signal strength. A wealth of recent studies is helping decipher how antigen persistence, TCR signal strength, and the cytokine milieu influence CD4+ T cell fate decisions, resulting in a fine balance between antiviral functions and tissue-sparing immunoregulation.

Advances in decoding antiviral TCR repertoires open the possibility of rapidly identifying protective TCR clonotypes, which may be harnessed in vaccination and immunotherapeutic approaches. Adoptive transfer of TCR engineered T cells is showing evidence of clinical benefit in the cancer field, for instance in the prevention of melanoma and acute myeloid leukemia relapse [187,188]. Transferred CD4+ T cells contribute to tumoricidal activity by direct killing of MHC II+ tumoral cells, providing help to cytotoxic CD8+ T cells, and also priming tumoricidal M1 macrophages [189,190]. Adoptive transfer of CMV-, Epstein–Barr virus-, and adenovirus-specific specific T cells is also used to prevent viral reactivation in immunocompromised patients [191], emphasizing the potential of TCR engineering for generating highly efficient antiviral T cells.

## Abbreviations

AIDS	Acuired Immunodeficiency Syndrome
AP-1	Activator Protein-1
APC	Antigen Presenting Cell
APOBEC3G	Apolipoprotein B mRNA editing enzyme, catalytic polypeptide-like 3G
Ascl-2	Achaete-scute complex homolog 2
BATF	Basic leucine zipper ATF-like transcription factor
Bcl-6	B-Cell Lymphoma-6
bNAb	Broadly Neutralizing Antibody
CCL3	Chemokine C-C Ligand-3
CD	Cluster of Differentiation
CMV	Cytomegalovirus
COVID-19	Coronavirus Disease -year 2019
CRTAM	Cytotoxic and Regulatory T Cell Molecule
CTLA-4	Cytotoxic T-Lymphocyte-Associated protein 4
CXCR5	C-X-C chemokine receptor type 5
DC	Dendritic Cells
EBI-2	Epstein-Barr virus-Induced Gene 2
GC	Germinal Center
HAV	Hepatitis A Virus
HCV	Hepatitis C Virus
HIV	Human Immunodeficiency Virus
IAV	Influenza A Virus
ICOS	Inducible T cell Costimulator
IDO	Indoleamine 2,3-dioxygenase
IFITM3	Interferon-induced transmembrane protein 3
IFN	Interferon
IL	Interleukin
IRF4	Interferon Regulatory Factor 4
ISG	Interferon-Stimulated Genes
JC polyomavirus	John Cunningham polyomavirus
LAG-3	Lymphocyte Activation gene-3
LCMV	Lymphocytic Choriomeningitis Virus
MAF	Musculoaponeurotic Fibrosarcoma oncogene
MHC	Major Histocompatibility Complex
MX2	MX Dynamin Like GTPase 2
NFAT2	Nuclear Factor of Activated T cells-2
NK	Natural Killer
PD-1	Programmed cell death-1
pMHC	Peptide-MHC complex
RSV	Respiratory Syncytial Virus
Runx3	Runt-related Transcription Factor-3
SARS-CoV-2	Severe Acute Respiratory Syndrome-related Coronavirus 2
SIV	Simian Immunodeficiency Virus
TCM	T Central Memory
TCF-1	T cell factor-1
TCR	T Cell Receptor
TF	Transcription Factor
Tfh	T follicular helper
Th	T helper
Treg	Regulatory T cell
TIGIT	T cell Immunoreceptor with Ig and ITIM Domains
TNF-α	Tumor Necrosis factor-alpha
TRM	T resident Memory
TGF-β	Transforming Growth Factor-beta
VSV	Vesicular Stomatitis Virus
VZV	Varicella Zoster Virus
